# Interaction of Bacterial Membrane Vesicles with Specific Species and Their Potential for Delivery to Target Cells

**DOI:** 10.3389/fmicb.2017.00571

**Published:** 2017-04-07

**Authors:** Yosuke Tashiro, Yusuke Hasegawa, Masaki Shintani, Kotaro Takaki, Moriya Ohkuma, Kazuhide Kimbara, Hiroyuki Futamata

**Affiliations:** ^1^Applied Chemistry and Biochemical Engineering Course, Department of Engineering, Graduate School of Integrated Science and Technology, Shizuoka UniversityHamamatsu, Japan; ^2^Graduate School of Science and Technology, Shizuoka UniversityHamamatsu, Japan; ^3^Japan Collection of Microorganisms, RIKEN BioResource CenterTsukuba, Japan; ^4^Department of Applied Chemistry and Biochemical Engineering, Faculty of Engineering, Shizuoka UniversityHamamatsu, Japan; ^5^Research Institute of Green Science and Technology, Shizuoka UniversityShizuoka, Japan

**Keywords:** membrane vesicles, DLVO theory, zeta potential, horizontal gene transfer, *Buttiauxella agrestis*

## Abstract

Membrane vesicles (MVs) are secreted from a wide range of microbial species and transfer their content to other cells. Although MVs play critical roles in bacterial communication, whether MVs selectively interact with bacterial cells in microbial communities is unclear. In this study, we investigated the specificity of the MV-cell interactions and evaluated the potential of MVs to target bacterial cells for delivery. MV association with bacterial cells was examined using a fluorescent membrane dye to label MVs. MVs derived from the enterobacterium *Buttiauxella agrestis* specifically interacted with cells of the parent strain but interacted less specifically with those of other genera tested in this study. Electron microscopic analyses showed that MVs were not only attached on *B. agrestis* cells but also fused to them. The interaction energy, which was characterized by hydrodynamic diameter and zeta potential based on the Derjaguin–Landau–Verwey–Overbeek (DLVO) theory, was significant low between MVs and cells in *B. agrestis*, compared to those between *B. agrestis* MVs and cells of other genera. Similar specific interaction was also occurred between *B. agrestis* MVs and cells of six other species belonging to *Buttiauxella* spp. *B. agrestis* harboring plasmid pBBR1MCS-1 secreted plasmid-containing MVs (p-MVs), and plasmid DNA in p-MVs was transferred to the same species. Moreover, antibiotic-associated MVs enabled effective killing of target species; the survival rate of *B. agrestis* was lower than those of *Escherichia coli* and *Pseudomonas aeruginosa* in the presence of gentamicin-associated MVs derived from *B. agrestis*. Altogether, we provide the evidence that MVs selectively interact with target bacterial cells and offer a new avenue for controlling specific bacterial species using bacterial MVs in microbial communities.

## Introduction

Many bacteria and archaea produce small bilayered particles (20–200 nm) from their surfaces (Mashburn-Warren and Whiteley, 2006; Deatherage and Cookson, 2012). These microbial liposomes are called membrane vesicles (MVs) or outer membrane vesicles (OMVs), particularly in gram-negative bacteria. MVs are composed of proteins, phospholipids, and polysaccharides and contain proteins, DNA, RNA and, in some cases, quorum sensing signals (Mashburn and Whiteley, 2005; Kulp and Kuehn, 2010; Toyofuku et al., 2014; Schwechheimer and Kuehn, 2015; Turnbull et al., 2016). MVs have multifunctional roles, such as transferring nucleic acids or toxic compounds to other cells, promoting the formation and maintenance of biofilm, releasing unnecessary compounds from cells, providing resistance to antibiotics and phages and extending the membrane for extracellular electron transport (Tashiro et al., 2012; Biller et al., 2014; Pirbadian et al., 2014; Schwechheimer and Kuehn, 2015; Toyofuku et al., 2015; Roier et al., 2016). Importantly, substances such as virulence factors and genetic materials are highly concentrated in MVs and remain stable against environmental stresses.

Microbes interact with each other in multicellular communities, and this interaction includes both cooperative and offensive functions for bacterial survival (Shank and Kolter, 2009; Okabe et al., 2010; Tashiro et al., 2013). MVs play important roles in microbial interactions, as they transfer their contents to other microbial cells, even over long distances. Several previous studies have shown that MVs are involved in DNA transfer among bacteria (Dorward et al., 1989; Kolling and Matthews, 1999; Yaron et al., 2000; Klieve et al., 2005; Chiura et al., 2011; Fulsundar et al., 2014; Ho et al., 2015). Gene transfer via MVs has attracted particular interest because DNA encapsulated by MVs is stable against DNase (Renelli et al., 2004), enabling the long-distance transport of cytoplasmic contents and resulting in horizontal gene transfer. Additionally, MVs package QS signals, such as *Pseudomonas* quinolone signals and hydrophobic homoserine lactones (Mashburn and Whiteley, 2005; Li et al., 2016). QS signals associated with MVs are transferred to bacterial cells and control QS-regulated gene expression (Tashiro et al., 2010). MVs also have roles in cell-cell inhibition and killing among competing species (Berleman and Auer, 2013). Lytic activities have been reported in MVs derived from *Pseudomonas aeruginosa, Shigella flexneri*, and *Myxococcus xanthus* (Kadurugamuwa and Beveridge, 1996, 1999; Kadurugamuwa et al., 1998; Evans et al., 2012). Thus, MVs play crucial roles in intra- and interspecies communication; however, in bacterial cell–cell interactions via MVs, the recipients of MVs are not determined or not fully understood (Hasegawa et al., 2015). Elucidating the selectivity in MV interactions with bacterial cells is critical for an improved understanding of bacterial interactions in communities.

Despite the fact that the targeted delivery of signaling and lytic molecules via MVs in microbial communities has not been fully characterized, several studies have indicated that MVs derived from pathogens enable the target delivery of the interior toxins to host cells (Kesty et al., 2004; Bauman and Kuehn, 2009; Parker et al., 2010). Transmembrane or other surface-exposed proteins encode specific peptide sequences and recognize target cells or tissues. The presence of specific peptides on the surface of MVs also enables cell targeting (Alves et al., 2015). For example, addition of the Ail peptide from *Yersinia pestis* to MVs derived from *Escherichia coli* promoted delivery of the MV contents to eukaryotic cells *in vitro* (Kesty and Kuehn, 2004), and *E. coli* MVs with an anti-HER2 affibody on their surface could selectively deliver their contents to tumor cells. Thus, engineering MVs involves investigating both outer membrane protein adhesion for the host cell interaction with MVs and developing strategies for utilizing MVs as nanomedicines to achieve a cell-specific drug delivery system.

In the current study, we investigated whether bacterial MVs deliver their content to specific bacterial cells. Understanding the selective delivery of MVs to target bacterial cells offers a new avenue for controlling bacterial cells in heterogeneous samples. As a result of this screening, we found that MVs derived from the enterobacterium *Buttiauxella agrestis* CUETM77-167 specifically interacted with the same species. We used this bacterium as a model organism and characterized the specific interaction of its MVs. First, we examined the electrostatic energy between MVs and cells and showed that there was a low electrostatic energy between them in *Buttiauxella* spp. Next, we found that MVs attached to bacterial cells and transferred their contents, including a plasmid, to bacterial cells. Finally, we investigated whether the specific interaction of MVs enabled them to deliver an antibiotic to control target cells.

## Materials and Methods

### Microbial Strains, Plasmids, and Primers

The microbial strains, plasmids, and primers used in this study are listed in **Table 1**. Microbial cells were grown in tryptic soy broth (TSB: Becton, Dickinson and Company, Franklin Lakes, NJ, USA) medium shaken at 200 rpm. *Buttiauxella* spp., *Corynebacterium glutamicum, Micrococcus luteus, Flavobacterium johnsoniae, Rhizobium* spp., and *Pseudomonas alcaligenes* were grown at 30°C. *Bacillus subtilis, Hydrogenophaga pseudoflava, E. coli, Erwinia persicina*, and *P. aeruginosa* were grown at 37°C. For genetic manipulations, LB (Luria-Bertani Lennox: 1% w/v tryptone, 0.5% yeast extract and 0.5% NaCl) was used. When necessary, chloramphenicol was used at a concentration of 20 μg/mL for *E. coli* and *B. agrestis*. *E. coli* β2163 was used for conjugation to transfer the plasmid pBBR1MCS-1 to *B. agrestis*. DAPA (2,6-diaminopimelic acid) was used at a concentration of 60 μg/mL in LB medium for the growth of *E. coli* β2163.

**Table 1:** Bacterial strains, plasmid, and primers used in this study.

Strains	Genotype or description	Reference
***Escherichia coli***		
MG1655	F- λ- *ilvG rfb-50 rph-1*	Laboratory collection
DH5α	*supE44 hsdR17*(r_K_^-^ m_K_^-^) *thi-1 recA1 gyrA96* (Nal^R^) *relA1* Δ*lac* (*lacIZYA*-*argF*) U169 *deoR* (ϕ80 *lacZ*ΔM15)	Laboratory collection
β2163	(F–) RP4-2-Tc::Mu *dapA*::(*erm-pir*) Km^R^ Em^R^	Demarre et al., 2005
***Buttiauxella* spp.**		
*B. agrestis* CUETM77-167	JCM 1090 (DSM 4586)	BRC-JCM RIKEN/DSMZ
*B. brennerae* S1/6-571	DSM 9396	DSMZ
*B. ferragutiae* CDC1180-81	DSM 9390	DSMZ
*B. noackiae* NSW11	DSM 9401	DSMZ
*B. izardii* S32-161	DSM 9397	DSMZ
*B. warmboldiae* NSW326	DSM 9404	DSMZ
*B. gaviniae* S1/1-984	DSM 9393	DSMZ
**Other strains**		
*Pseudomonas aeruginosa* PAO1	Type strain	Holloway et al., 1979
*Corynebacterium glutamicum* AJ224	JCM 1308	BRC-JCM RIKEN
*Micrococcus luteus* JCM 1464	JCM 1464	BRC-JCM RIKEN
*Bacillus subtilis* C1	JCM 1103	BRC-JCM RIKEN
*Flavobacterium johnsoniae* JCM 8514	JCM 8514	BRC-JCM RIKEN
*Rhizobium halotolerans* JCM 17536	JCM 17536	BRC-JCM RIKEN
*Rhizobium soli* DS-42	JCM 14591	BRC-JCM RIKEN
*Hydrogenophaga pseudoflava* GA3	JCM 21410	BRC-JCM RIKEN
*Erwinia persicina* HK204	JCM 3704	BRC-JCM RIKEN
*Pseudomonas alcaligenes* JCM 20561	JCM 20561	BRC-JCM RIKEN
**Plasmid**		
pBBR1MCS-1	Broad-host range vector, Cm^R^ *lacZα rep mob*	Kovach et al., 1995
**Primers**		
CamR-f	5′-TTCCACACAACATACGAGCCG-3′	This study
CamR-r	5′-CATTATGCAGCTGGCACGAC-3′	This study

### Vesicle Extraction and Purification

Membrane vesicle extraction and purification were carried out as previously described (Tashiro et al., 2009). To obtain a sample of MVs without bacterial cells, 100 mL of overnight batch culture was centrifuged for 15 min at 6,000 × *g*, 4°C. The supernatant was filtered through 0.45 and 0.20 μm membrane filters and ultracentrifuged for 2 h at 100,000 × *g* and 4°C using an angle rotor (P45AT, Hitachi, Tokyo, Japan). The pellets were resuspended in 200 μL of 50 mM HEPES-0.85% NaCl (HEPES-NaCl buffer). For MV purification, MVs were labeled with 100 μg/mL FM4-64 in HEPES-NaCl buffer and washed using ultracentrifugation. MV samples were adjusted to 1 mL of 45% (w/v) iodixanol (OptiPrep; Axis-Shield Diagnostics Ltd., Dundee, UK) in HEPES-NaCl, transferred to the bottom of ultracentrifuge tubes, and layered with iodixanol-HEPES-NaCl (2 mL of 40, 35, 30, 25, and 20%). The samples were ultracentrifuged for 3 h at 100,000 × *g* and 4°C using a swing rotor (P40ST, Hitachi). Then, 500 μL fractions were collected from each gradient. To confirm the fraction containing MVs, the phospholipid and protein concentrations in each fraction were measured using the Stewart assay and the bicinchoninic acid (BCA) protein assay. The fraction containing MVs was ultracentrifuged and resuspended in HEPES-NaCl.

### Quantification of Vesicle Production

Membrane vesicle production was quantified by determining the phospholipid concentration in the bacterial culture supernatant. The phospholipid concentration was measured using a previously reported method with some modifications (Stewart, 1980). Briefly, 1 mL of the supernatant filtered through a 0.20-μm membrane, 200 μL of concentrated ammonium ferrothiocyanate solution (135 g/L iron (III) chloride hexahydrate and 152 g/L ammonium thiocyanate), and 200 μL of chloroform were vortexed. The absorption of the chloroform layer (lower layer) was measured at 488 nm. To construct a calibration curve, L-α-phosphatidylethanolamine was used as a reference standard. MV production was normalized to the quantity of cell protein. Bacterial cells were lysed by 5% SDS, and the protein concentration was determined by a BCA protein assay (Pierce BCA Protein Assay Kit; Thermo, Rockford, IL, USA).

### Vesicle Association Assay

The MV association degree was determined by the relative fluorescence units (RFUs) of fluorescently labeled MVs per concentration of cell protein. The extracted MVs (200 μg phospholipid) were incubated with 5 μg/mL FM4-64 in 1 mL of phosphate-buffered saline (PBS) for 30 min at 30°C and washed three times in PBS. In some cases, 100 μg/mL fluorescein isothiocyanate (FITC) or 5 μM DiO was used instead of FM4-64. Bacterial cells grown to the late exponential phase were washed in PBS and diluted to an OD_600_ of 1.0. FM4-64-labeled MVs (20 μg of phospholipid) were incubated with 1 mL of bacterial cells (OD_600_ of 1.0) for 30 min at 30°C. Bacterial cells with FM4-64-labeled MVs were washed in PBS. The fluorescence of FM4-64 was measured using a microplate reader (Infinite M200; TECAN, Männedorf, Switzerland), and the cell protein concentration was measured by a BCA protein assay.

### Flow Cytometry Analysis

Flow cytometry analysis using an EPICS Altra AJ49030 system (Beckman Coulter, Brea, CA, USA) was performed to detect cells associated with fluorescence-labeled MVs. MVs (200 μg of phospholipid) were labeled with FM4-64 and washed in PBS by ultracentrifugation. Bacterial cells were adjusted to OD_600_ = 1.0 in PBS, labeled with 100 μg/mL FITC for 30 min at 30°C and washed three times in PBS. FM4-64-labeled MVs and FITC-labeled bacterial cells were incubated for 30 min at 30°C and washed with PBS three times. The particle number and fluorescence intensity of bacterial cells associated with MVs were analyzed by flow cytometry.

### Microscopy Observation

For fluorescence microscopy analysis, the cells were examined using the fluorescence microscope OLYMPUS BX53 (OLYMPUS, Tokyo, Japan), and images were taken with the CCD camera DP72 (OLYMPUS) and the imaging software CellSens (OLYMPUS). Bacterial cells were adjusted to OD_600_ = 1.0 in PBS, labeled with 100 μg/mL FITC for 30 min at 30°C and washed three times in PBS. FM4-64-labeled MVs and FITC-labeled bacterial cells were incubated for 30 min at 30°C and washed with PBS three times.

For transmission electron microscopy observations of purified MVs, MVs were placed on Cu400 mesh grids, stained with 2% uranyl acetate and visualized by JEM100EX (JEOL, Tokyo, Japan) by the Hanaichi Ultrastructure Research Institute (Okazaki, Aichi, Japan). For other TEM analyses, the samples were placed on Cu200 mesh grids that had been treated with 0.01% poly-L-lysine in PBS. The grids were rinsed with distilled water and stained with 2% phosphotungstic acid neutralized with KOH. After rinsing with distilled water and dried, the samples were visualized by JEM 2000FX-II (JEOL).

### Immunogold Labeling

The extracted MVs were labeled in 100 μg/mL FITC and washed with PBS by ultracentrifugation. Bacterial cells were adjusted to OD_600_ = 1.0 in PBS, incubated with FITC-labeled MVs for 30 min at 30°C and washed with PBS three times. The samples were placed on Cu200 mesh grids that had been treated with 0.01% poly-L-lysine in PBS. The bacteria were fixed with 4% paraformaldehyde in PBS for 30 min and washed with PBS. The specimens were incubated with 1% bovine serum albumin (BSA) in PBS for 5 min and washed again with PBS. The specimens were then incubated with anti-FITC antibody (Sigma–Aldrich) at a dilution of 1:300 in PBS containing 1% BSA for 60 min. The specimens were washed with PBS, incubated with colloidal gold-labeled anti-rabbit immunoglobulin (diameter of colloidal gold particles, 15 nm) at a dilution of 1:1000 in PBS containing 1% BSA for 60 min, and then washed. The grids were fixed with 2% glutaraldehyde in PBS for 15 min and washed with PBS. After being stained with 2% phosphotungstic acid and washed, the samples were observed by JEM 2000FX-II.

### Physicochemical Analysis of Particles

The particle size distribution and zeta potential were analyzed with a Zetasizer Nano ZS particle analyzer (Malvern Instruments, Malvern, UK) at 25°C. Purified MVs were adjusted to a concentration of 20 μg of phospholipid/mL in PBS. Cells grown to the stationary phase in TSB were washed with PBS and adjusted to OD_600_ = 0.1. The hydrodynamic zeta average diameter was calculated by the light scattering method, and the zeta potential was determined by applying the Smoluchowski approximation. The electrostatic charge on the surfaces of MVs and cells was characterized as the zeta potential.

### Calculating the Interaction Energy between Cells and Vesicles

The potential interaction energy between cells and vesicles was calculated based on the Derjaguin–Landau–Verwey–Overbeek (DLVO) theory. The interaction energy *V*_total_ was defined as:

(1) $$V_{\text{total}}=V_{\text{A}}+V_{\text{R}}$$

where *V*_A_ is an attractive London-van der Waals interaction, and *V*_R_ is an electric repulsive interaction energy. Based on previous studies (Ohki and Ohshima, 1999), *V*_A_ was defined as follows:

(2) $$V_{\text{A}}=-\frac{A}{6}\{\frac{2aa^{\prime }}{R^{2}-{\left(a+a^{\prime }\right)}^{2}}+\frac{2aa^{\prime }}{R^{2}-{\left(a-a^{\prime }\right)}^{2}}+\ln\frac{R^{2}-{\left(a+a^{\prime }\right)}^{2}}{R^{2}-{\left(a-a^{\prime }\right)}^{2}}\}$$

where

(3) $$R=r+a+a^{\prime }$$

where *A* is the Hamaker constant; *a* and *a*′ are the radii of the cells and vesicles, respectively; *R* is the separation distance between the center of the particles of cells and vesicles; and *r* is their separation distance. Hamaker constant *A* is described as follows:

(4) $$A=\pi^{2}q^{2}\lambda$$

where *q* is the volume density, and λ is London-van der Waals constant. *A* of the phospholipid bilayer was expressed as 4 × 10^-19^ N (Ohki et al., 1982).

According to a previous report (Ohki and Ohshima, 1999), *V*_R_ was defined as follows:

(5) $$V_{\text{R}}=\frac{2\pi \pi_{\text{r}}\varepsilon_{0}aa^{\prime }(\phi^{2}+\phi^{\prime }{}^{2})}{aa^{\prime }}\left[\frac{2\phi \phi^{\prime }}{\phi^{2}+\phi^{\prime }{}^{2}}\ln\frac{1+\exp(-\kappa \text{r})}{1-\exp(-\kappa \text{r})}+\ln\{1-\exp(-2\kappa \text{r})\}\right]$$

where φ and φ′ are the Stern potentials of cells and vesicles, respectively, and κ is the Debye constant. The values of the Stern potentials were used as zeta potentials (ζ) obtained by the experiments. When the absolute values of φ and φ′ were less than 60 mV (κr > 1), *V*_R_ was approximated as follows:

(6) $$V_{\text{R}}=2\varepsilon_{\text{r}}\varepsilon_{\text{0}}\kappa exp(-\kappa r)\{\text{2}\phi \phi^{\prime }\text{-}(\phi^{\text{2}}\text{+}\phi^{\prime }{}^{\text{2}})\text{exp}(\text{-}\kappa \text{r})\}$$

where 𝜀_r_ is the relative permittivity for the medium, and 𝜀_0_ is the permittivity of a vacuum. The Debye constant κ was expressed by the following:

(7) $$\kappa ={\left(\frac{2000e^{2}N_{\text{A}}cz^{2}}{\varepsilon_{\text{r}}\varepsilon_{0}kT}\right)}^{\frac{1}{2}}$$

where *e* is the elementary charge, *N*_A_ is Avogadro’s number, *c* is the ion concentration, *z* is the charge of ions and *k* is the Boltzmann constant.

### Vesicle-mediated Gene Transfer

Experiments for vesicle-mediated gene transfer were based on a previously published method (Yaron et al., 2000). The donor strain, *B. agrestis* CUETM77-167/pBBR1MCS-1, was grown in LB medium containing chloramphenicol at 200 rpm for 16 h at 30°C. MVs were extracted using sterile ultracentrifuge tubes after filtering the supernatant. Extracted MVs were reacted with DNase I in reaction buffer [40 mM Tris-HCl (pH 7.9), 10 mM NaCl, 6 mM MgCl_2_, and 1 mM CaCl_2_], and the extracellular DNA around the MVs was degraded. Then, the MVs were washed with PBS by ultracentrifugation. MVs (20 μg of phospholipids) and approximately 1,000 recipient bacterial cells of *B. agrestis* were mixed in 1 mL of PBS, and the cells were washed with PBS at each time point. The transfer of plasmid DNA was examined by counting the colony forming units (CFUs) on agar medium containing chloramphenicol. As a control experiment, naked plasmid DNA, which was extracted from *B. agrestis* CUETM77-167/pBBR1MCS-1, was added to the cell suspension (final DNA concentration was 10 ng/mL), and the possibility of natural transformation was examined by CFU counting. PCR analysis with a primer pair (CamR-f and CamR-r, listed in **Table 1**), and plasmid extraction were conducted to confirm whether the obtained colonies harbored the plasmid pBBR1MCS-1.

### Real-time PCR Analysis

The copy number of the plasmid was determined using real-time PCR analysis. The chloramphenicol acyl transferase gene in pBBR1MCS-1 was amplified and quantified using LightCycler FastStart DNA Master SYBR Green I (Roche, Basel, Switzerland) with a primer pair (CamR-f and CamR-r) on a LightCycler 2.0 (Roche) according to the instruction manual.

### Nanoparticle Tracking Analysis

The concentration of MVs was measured by nanoparticle tracking analysis using a NanoSight LM10 instrument (Malvern) equipped with sCMOS camera (Andor, Belfast, UK). The samples were diluted to an average concentration of 10^6^–10^9^ particles per milliliter in 50 mM HEPES buffer. Five replicates videos were collected from each sample, and particle movement was analyzed by NTA software (Version 3.1, Malvern). The velocity of particle movement was used to calculate the particle size using the two-dimensional Stokes-Einstein equation.

### Vesicle-Mediated Antibiotic Transfer

Gentamicin-containing MVs (g-MVs) were prepared as previously described (Kadurugamuwa and Beveridge, 1995). Briefly, *B. agrestis* CUETM77-167 was grown in TSB medium until the late stationary phase, and gentamicin was added to a final concentration of 32 μg/mL [four times the minimal inhibitory concentration (MIC) of gentamicin for this strain]. After a further 30 min of cultivation, MVs were extracted from the bacterial culture. The amount of gentamicin in the MV suspension was determined by examining the bacterial killing effect of homogenized MVs. The concentration of gentamicin in MVs was calculated using the number of MVs in the MV suspension. To analyze the killing effect on a single bacterial species, MVs (20 μg of phospholipids) and approximately 1,000 bacterial cells were mixed in 1 mL of PBS, and the cells were washed with PBS at each time point. The surviving cells were counted by CFU. To analyze the killing effect in mixed bacterial culture, MVs (20 μg of phospholipids) and approximately 1,000 cells of each bacterial species (*B. agrestis* CUETM77-167 and *E. coli* DH5α) were mixed in 1 mL of PBS, and the cells were washed with PBS at each time point. The surviving cells were counted by CFU on an agar plate containing 200 μg/mL 5-bromo-4-chloro-indolyl β-D-galactopyranoside (X-gal) and 0.5 mM isopropyl β-D-1-thiogalactopyranoside (IPTG). *B. agrestis* and *E. coli* DH5α showed blue and white colonies, respectively.

### Determination of the Antibiotic Concentration in Vesicles

Vesicles associated with gentamicin were homogenized by an ultrasonic disintegrator. The suspended lysed vesicles containing gentamicin were diluted and added to LB agar on a 24-well plate before the agar had gelatinized. A cell suspension of *E. coli* MG1655 was grown on the plate, and the result was compared to that on an agar plate containing gentamicin. Using the MIC, the gentamicin concentration in the sample was determined. The vesicle particle number and average particle size were measured by nanoparticle tracking analysis, and the gentamicin concentration in the vesicle particle was calculated.

### Phylogenetic Analysis

Sequence data were accessed from NCBI (GenBank accession numbers are AJ233400 to AJ233406 and NC_000913). 16S rRNA gene sequences were aligned using the ClustalW program, and distance matrix trees were constructed by the neighbor-joining method. The topology of the trees was evaluated by bootstrapping with 1,000 replicates, and the percentages of values are shown on branching points.

### Statistical Analysis

Error bars throughout the figures refer to the standard error for all of the experiments. The statistical significance of the differences between groups was examined using unpaired and one-way Student’s *t*-tests. A *P*-value of <0.005 was considered to indicate statistical significance.

## Results

### Specific Interaction of Vesicles with Bacterial Cells in *B. agrestis*

**FIGURE 1:**
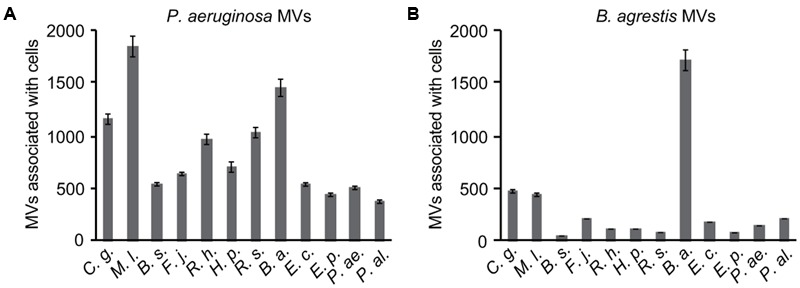
**Association of membrane vesicles (MVs) with various bacterial species.** MVs (20 μg/mL of phospholipids) derived from *P. aeruginosa* PAO1 **(A)** and *B. agrestis* CUETM77-167 **(B)** were labeled with FM4-64 and incubated with bacterial cells for 30 min at 30°C. *Corynebacterium glutamicum* AJ2247 (*C. g.*), *Micrococcus luteus* JCM 1464 (*M. l.*), *Bacillus subtilis* C1 (*B. s.*), *Flavobacterium johnsoniae* JCM 8514 (*F. j.*), *Rhizobium halotolerans* JCM 17536 (*R. h.*), *R. soli* DS-42 (*R. s.*), *Hydrogenophaga pseudoflava* GA3 (*H. p.*), *Buttiauxella agrestis* CUETM77-167 (*B. a.*), *Escherichia coli* MG1655 (*E. c.*), *Erwinia persicina* HK204 (*E. p.*), *Pseudomonas aeruginosa* PAO1 (*P. ae.*), and *P. alcaligenes* JCM 20561 (*P. al.*) were used as the recipient strains. The MVs associated with the cells were identified by RFUs normalized to the cellular protein concentration (mg/mL). The data are shown as the mean ± standard deviations from three replicates.

To identify putative high MV-producing bacteria, we examined the phospholipid concentrations in the supernatant of 76 stationary cultures of microbial strains in laboratory stocks. We arbitrarily selected eleven bacterial strains, including *C. glutamicum* AJ2247, *M. luteus* JCM 1464, *B. subtilis* C1, *F. johnsoniae* JCM 8514, *R. halotolerans* JCM 17536, *R. soli* DS-42, *H. pseudoflava* GA3, *B. agrestis* CUETM77-167, *E. persicina* HK204, *P. aeruginosa* PAO1, and *P. alcaligenes* JCM 20561, which are supposed to release MVs in the culture at high levels compared to *E. coli* MG1655 (Supplementary Figure S1), for use in further analyses. Each supernatant from the bacterial culture was ultracentrifuged, and the pellet was labeled with the styryl dye FM4-64, a lipophilic styryl compound that stains the vacuolar membrane. To determine whether each putative MV selectively interacted with bacterial cells, MVs associated with bacterial cells were quantified by fluorescence detection (Supplementary Table S1). The interaction of MVs derived from *P. aeruginosa* with bacterial cells has been studied well (Kadurugamuwa and Beveridge, 1996, 1997; Li et al., 1998; Tashiro et al., 2010) and showed different levels of association based on bacterial species in this study (**Figure 1A**). Notably, MVs derived from *B. agrestis* CUETM77-167 interacted with the parent strain at a significant level but not with other cells (**Figure 1B**). This specific interaction was not due to the labeling of FM4-64 because similar results were obtained when MVs from *B. agrestis* were labeled with amino group-reactive FITC or carbocyanine membrane dye DiO (Supplementary Figure S2).

**FIGURE 2:**
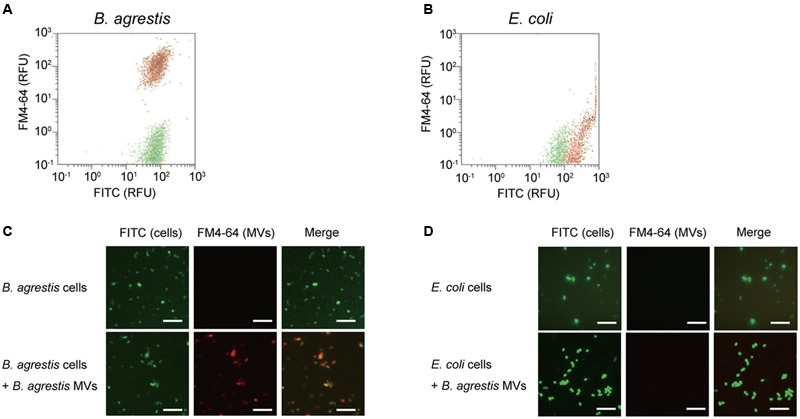
**Detection and observation of the association of MVs derived from *B. agrestis* CUETM77-167 with CUETM77-167 and *E. coli* MG1655 cells.** FM4-64-labeled MVs (20 μg/mL of phospholipids) were incubated with FITC-labeled bacterial cells for 30 min at 30°C. **(A,B)** Flow cytometry analysis of CUETM77-167 **(A)** and MG1655 cells **(B)** associated with MVs. FITC-labeled cells (green plots) and FITC-labeled cells incubated with FM4-64-labeled MVs (red plots) underwent flow cytometry independently, and the plots are shown in the same chart. **(C,D)** Fluorescence microscopy observation of FITC-labeled CUETM77-167 **(C)** and MG1655 **(D)** cells incubated with FM4-64-labeled MVs derived from CUETM77-167. The bars indicate 5 μm.

To further corroborate the specific interaction of MVs derived from *B. agrestis* with the same species, the association between MVs and bacterial cells was analyzed at the single-cell level. *B. agrestis* cells labeled with FITC were incubated with FM4-64-labeled MVs for 30 min, and washed cells were analyzed by flow cytometry. As a control, FITC-labeled cells, which were not treated with MVs, were also assessed by flow cytometry. The fluorescence intensities of both FM4-64 and FITC were detected in MV-reacted cells (approximately 1 × 10^2^ RFU), while only the intensity of FITC was detected in the control sample (**Figure 2A**). Then, the same experiment was conducted using *E. coli* and MVs derived from *B. agrestis*. The fluorescence of FM4-64 was not detected in *E. coli* either with or without *B. agrestis* MVs (**Figure 2B**). Similar results were also obtained by fluorescence microscopy analysis; the fluorescence of both FM4-64 and FITC was observed in MV-treated *B. agrestis* (**Figure 2C**), but the fluorescence of FM4-64 was not observed in *E. coli*, which was treated identically (**Figure 2D**). These data confirm the hypothesis that MVs derived from *B. agrestis* specifically interact with bacterial cells of the same species.

To determine if bacterial viability is essential for MV-cell interactions, the associations of MVs with viable and dead cells were examined. Ultraviolet (UV)-treated cells showed a high association with *B. agrestis* MVs as well as non-UV treated cells (Supplementary Figure S3), suggesting that the bacterial viability of recipients is not required for the specific MV-cell interaction in *B. agrestis*.

**FIGURE 3:**
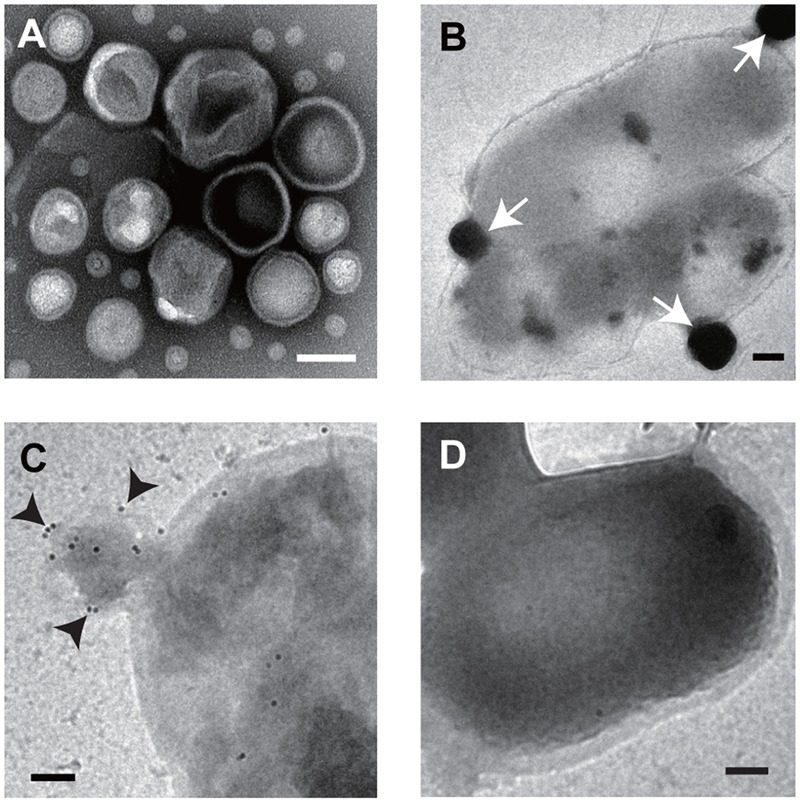
**TEM imaging. (A)** Image of purified MVs derived from *B. agrestis* CUETM77-167. **(B)** Association of FM4-64-labeled MVs with cells. The white arrows indicate MVs, which have a high density due to FM4-64 labeling. **(C)** Association of FITC-labeled MVs with cells. Cell-associated MVs were detected by small gold particles (black arrows) through the FITC antibody. **(D)** Cells with no addition of MVs were reacted with FITC antibody. All bars indicate 100 nm.

We confirmed that the extract from the supernatant of *B. agrestis* culture from ultracentrifugation contained spherical MVs by TEM observation (**Figure 3A**). Next, we investigated if supplemented MVs interacted with bacterial cells using TEM observation. FM dyes have been used to determine the localization of supplemented vesicles because their photoactivation creates reactive oxygen species, which form an electron-dense precipitate in the vacuoles that is readily visualized under electron microscope (Harata et al., 2001). Then, FM4-64-labeled MVs were incubated with non-labeled cells of *B. agrestis*, and TEM analysis was conducted to observe the interaction of added MVs with *B. agrestis* cells. The result showed that MVs attached to the cellular surface were darkly stained (**Figure 3B**), suggesting that additional MVs were definitely associated with cells of *B. agrestis*. To further analyze MV-cell interaction, FITC-labeled MVs were incubated with non-labeled cells, and FITC was labeled by gold particles using a gold-conjugate antibody against FITC. The fusion of MVs onto bacterial cells was visualized, and MVs were labeled with gold particles (**Figure 3C**), suggesting that the MVs in the image were not those being released from cells but rather those being incorporated into cells. When MVs were not added to bacterial suspension before immunolabeling as the control, dense gold particles were not observed around cells (**Figure 3D**). Thus, the interaction of MVs with bacterial cells is not only the surface attachment but also the membranous fusion.

### Interaction Energy between Vesicles and Cells

**FIGURE 4:**
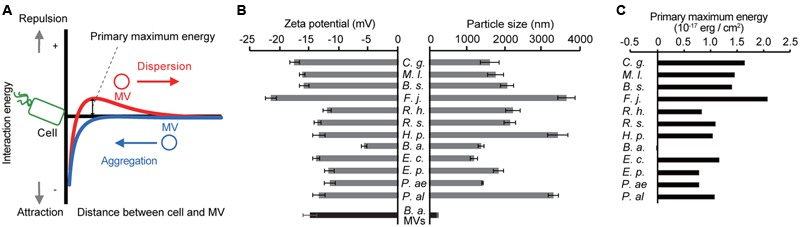
**Interaction energy between cells and MVs derived from *B. agrestis*. (A)** Model for the free energy profile of the interaction between cells and MVs according to a generalized Derjaguin–Landau–Verwey–Overbeek (DLVO) theory. When the energy barrier is large enough (red line), MVs are prevented from approaching cells by electrostatic repulsion. However, a small energy barrier causes MVs to aggregate with cells (blue line) due to van der Waals attractions. **(B)** Zeta potential and particle size of each bacterial cell (gray) and *B. agrestis* MVs (black). *C. g., Corynebacterium glutamicum* AJ224; *M. l., Micrococcus luteus* JCM 1464; *B. s., Bacillus subtilis* C1; *F. j., Flavobacterium johnsoniae* JCM 8514; *R. h., Rhizobium halotolerans* JCM 17536; *R. s., R. soli* DS-42; *H. p., Hydrogenophaga pseudoflava* GA3; *B. a., Buttiauxella agrestis* CUETM77-167; *E. p., Erwinia persicina* HK204; *P. ae., Pseudomonas aeruginosa* PAO1; *P. al., P. alcaligenes* JCM 20561. The data are shown as the mean ± standard deviation from three replicates. **(C)** Primary maximum energy between each cell and *B. agrestis* MVs based on the DLVO theory.

Because the specific interaction between MVs and cells occurred regardless of the viability of recipient cells, we hypothesized that the physicochemical properties of surfaces affect this association. When MVs and bacterial cells are assumed to be colloid particles, the interaction between two particles is explained by the DLVO theory (Hermansson, 1999; Ohki and Ohshima, 1999; Strevett and Chen, 2003). When the primary maximum energy is positive, there is an energy barrier between MVs and cells (**Figure 4A**). The interaction energy is the total of the van der Waals’ force and electric repulsion energy, and these depend on the particle size and zeta potential of the particles, respectively. To evaluate the interaction energy between MVs and bacterial cells, their surface thermodynamic properties were measured using a Zeta Sizer (**Figure 4B**). The interaction energy between *B. agrestis* MVs and each bacterial cell was calculated based on the DLVO theory (Supplementary Figure S4). Interestingly, the primary energy in the interaction of *B. agrestis* MVs with the same species was significantly lower than that of other bacterial cells (**Figure 4C**). These results suggest that one factor in the specific interaction of MVs with the same species in *B. agrestis* is the low interaction energy based on the DLVO theory.

### Specific Vesicle-cell Interaction Is Conserved in *Buttiauxella* spp.

**FIGURE 5:**
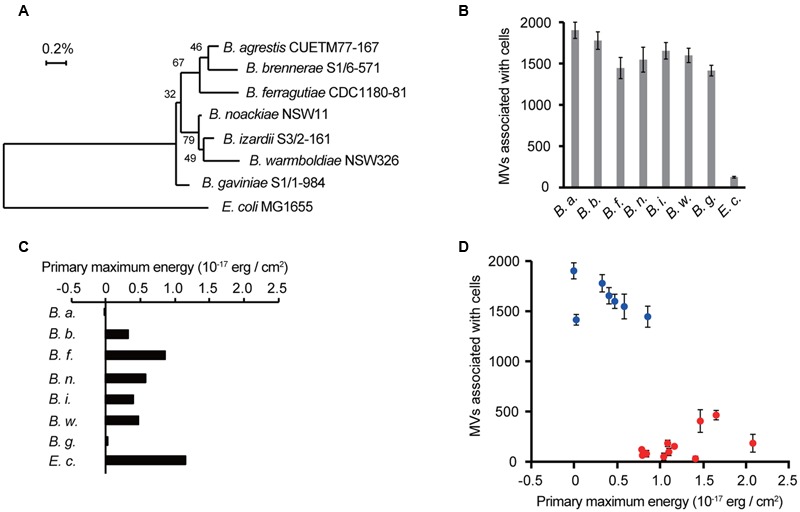
**Selective interactions between cells and MVs are conserved in *Buttiauxella* spp. (A)** The phylogenetic relationship between *Buttiauxella* spp. and *E. coli*. Numbers on the branching points are the percentages of bootstrap values with 1000 replicates. **(B)** Association of MVs derived from *B. agrestis* CUETM77-167 with bacterial cells. FM4-64-labeled MVs (20 μg/mL of phospholipids) were incubated with bacterial cells for 30 min at 30°C. MVs associated with cells were identified by RFUs of FM4-64 normalized to the cellular protein concentration (mg/mL). The data are shown as the mean ± standard deviation from three replicates. **(C)** Primary maximum energy between each cell and *B. agrestis* MVs. The values were calculated from the zeta potential and particle size based on the DLVO theory. **(D)** Relationship between the MV association with cells and primary maximum energy. Blue plots show *Buttiauxella* strains (from **B,C**), and red plots show other bacterial strains (from **Figures 1B, 4C**). The coefficient of determination *R*^2^ is 0.59.

Although a specific interaction of MVs with the same species was observed in *B. agrestis*, it was unknown whether this characteristic was confined to this bacterium. We prepared 6 other *Buttiauxella* strains (*B. brennerae* S1/6-571, *B. ferragutiae* CDC1180-81, *B. noackiae* NSW11, *B. izardii* S3/2-161, *B. warmboldiae* NSW326, and *B. gaviniae* S1/1-984), and the phylogenetic relationship among them and *E. coli* is shown in **Figure 5A**. Interestingly, the MVs derived from *B. agrestis* CUETM77-167 showed a much higher association with cells of *Buttiauxella* spp. than with *E. coli* (**Figure 5B**), suggesting that the specific interaction of MVs with cells is conserved in *Buttiauxella* spp. To determine whether the high interaction between MVs derived from CUETM77-167 and *Buttiauxella* strains is due to the physicochemical interaction energy, the zeta potentials and hydrodynamic diameters of each cell were examined. The calculated interaction energy based on the DLVO theory showed that the primary maximum energies of several *Buttiauxella* cells with MVs derived from CUETM77-167 were significantly lower than that for *E. coli* (**Figure 5C** and Supplementary Figure S5). We next evaluated the relationship between the primary maximum energies calculated by DLVO theory and the association values of MVs with bacterial cells. **Figure 5D** shows that plots of *Buttiauxella* strains are localized at different positions from those of other strains. Statistical analyses indicated significant differences between *Buttiauxella* strains and other strains in both association with *B. agrestis* MVs and primary maximum energies (Supplementary Figure S6), suggesting that interaction energy based on the DLVO theory is one factor explaining why MVs specifically interact with bacterial cells of *Buttiauxella* spp. However, the correlation between the two parameters is not linear in **Figure 5D** (the coefficient of determination *R*^2^ is 0.59), and other factors besides low interaction energy based on the DLVO theory may affect the specific interaction of MVs in *Buttiauxella* strains. When MVs and/or cells were treated with proteinase K, the association between MVs and cells in *B. agrestis* was decreased more than 50% (Supplementary Figure S7), suggesting that proteins localized on the surface of cells and MVs also affect the MV-cell interaction in *B. agrestis*.

### Vesicle-mediated DNA Transfer

**FIGURE 6:**
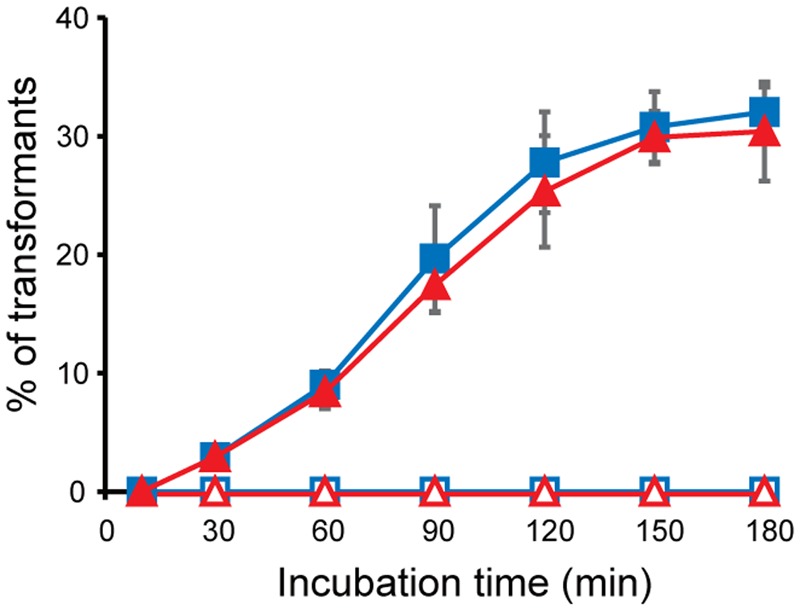
**Plasmid transfer through MVs derived from *B. agrestis*.** MVs were extracted from the culture of CUETM77-167 (open) or CUETM77-167/pBBR1-MCS (closed). DNase-treated (red triangles) or non-treated (blue squares) MVs (20 μg/mL of phospholipids) were incubated with CUETM77-167 at 30°C. Transformants were calculated by counting the CFUs on agar medium containing chloramphenicol. The data are shown as the mean ± standard deviation from three replicates.

To determine whether the interaction of MVs with bacterial cells contributes to the delivery of the MV contents to bacterial cells, we evaluated vesicle-mediated plasmid DNA transfer. *B. agrestis* harboring pBBR1MCS-1 was grown in TSB medium to the stationary phase, and plasmid-containing MVs (p-MVs) were extracted from the supernatant. An examination of the pBBR1MCS-1 concentration by quantitative PCR showed 3.11 × 10^6^ and 2.27 × 10^6^ copies/mL in the supernatant before and after the removal of MVs through ultracentrifugation, respectively, suggesting that at least approximately one-third of the plasmid localized in the extracellular milieu was associated with p-MVs in the *B. agrestis* supernatant. The external DNA surrounding p-MVs was degraded by DNase I treatment, and the plasmid concentration in the p-MVs was calculated by counting the number of MVs using nano tracking analysis. The results showed that p-MVs contain 1.03 × 10^9^ copies/mL of pBBR1MCS-1, indicating that p-MVs maintain a high concentration of plasmid and that the DNA in p-MVs was stable against DNase I treatment. When *B. agrestis* cells (approximately 1.0 × 10^3^ cells/mL) were treated with an excessive amount of p-MVs, more than 30% *B. agrestis* transformants were obtained after 3 h of incubation with DNase I-treated p-MVs and non-treated p-MVs (**Figure 6**), suggesting that the plasmid contained in MVs was transferred to bacterial cells. When naked plasmid DNA instead of p-MVs was added to directly bacterial cell suspension in this experiment (data not shown), indicating that natural transformation was not occurred in the condition. Notably, other methods for DNA transformation into this strain have not yet been established in our experiments, suggesting that DNA transfer via p-MVs is a useful tool to obtain transformants in this strain.

### Vesicles Enable the Selective Delivery of an Antibiotic

**FIGURE 7:**
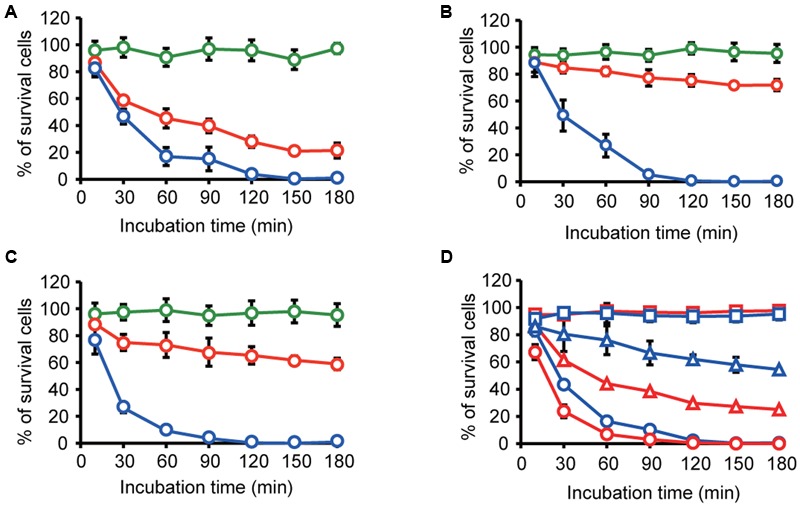
**Transfer of gentamicin through MVs derived from *B. agrestis*. (A–C)** Survival rate of *B. agrestis* CUETM77-167 **(A)**, *E. coli* MG1655 **(B)** or *P. aeruginosa* PAO1 **(C)**. Each bacterial cell was incubated with normal MVs (n-MVs: green), gentamicin-harboring MVs (g-MVs: red) and homogenized g-MVs (blue). The data are shown as the mean ± standard deviation from three replicates. **(D)** Survival rate of bacterial cells in the mixture of CUETM77-167 (red) and *E. coli* DH5α (blue). The samples were incubated with n-MVs (squares), g-MVs (triangles), and homogenized g-MVs (circles). The data are shown as the mean ± standard deviation from three replicates.

We expected that the specific interaction of MVs with bacterial cells could be used for the selective control of bacterial cells. Exposure to the antibiotic gentamicin has been shown to increase MV formation, and gentamicin was retained within the MVs (Kadurugamuwa and Beveridge, 1995; Fulsundar et al., 2014). We evaluated the potential of MVs as tools for the delivery of gentamicin to specific bacterial cells. *B. agrestis* was grown to the stationary phase, and gentamicin was added to the culture at a final concentration of 32 μg/mL (four times the MIC). MVs were extracted, and gentamicin was concentrated in MVs (146 μg/mL) based on the calculated gentamicin concentration and the number of MVs. We prepared gentamicin-associated MVs (g-MVs) and homogenized g-MVs, in which the gentamicin concentration was likely the same as that in the sample of g-MVs. As a negative control, MVs derived from *B. agrestis* not associated with gentamicin (n-MVs) were also prepared. These MVs were incubated with cells of *B. agrestis, E. coli*, and *P. aeruginosa*, and the survival rates were examined. The killing effect of g-MVs on *B. agrestis* was much greater than that on *E. coli* and *P. aeruginosa*, while homogenized g-MVs had a high killing effect on all of the bacteria that were tested in this experiment (**Figures 7A–C**). Under these experimental conditions, the gentamicin concentration of each sample ranged from 1.3 to 2.3 μg/mL, and this concentration was lower than the MIC of each strain. Subsequently, to determine whether selective antibiotic delivery via MVs occurred in the microbial complexes, the effect of g-MVs in the mixed samples containing *B. agrestis* and *E. coli* cells was examined. The results showed that the killing effect of g-MVs on *B. agrestis* was higher than that on *E. coli*, even in mixed samples (**Figure 7D**). These data indicate that the specific interaction of *B. agrestis* MVs is useful for killing target species in heterogeneous samples.

## Discussion

Although bacterial cell-cell interaction via MVs has attracted particular attention in the past decade, whether bacterial cells are recipients for secreted MVs is unclear. To our knowledge, this study is the first report that MVs secreted from a certain bacterial species selectively interact with specific cells. The main finding of this study is that MVs have a function of specific delivery of MV contents to intraspecies bacterial cells in *Buttiauxella* spp. *Buttiauxella* strains have been isolated from unpolluted soil and drinking water, surface water, sewage, soil, and fecal samples (Müller et al., 1996), but little is understood about their ecological characteristics in the environment. Although *Buttiauxella* spp. can be a host of plasmid pBP136 in soil bacteria (Shintani et al., 2014), MV production has not been studied in this bacterium.

One important factor in the association between MVs and bacterial cells is electric charges on the cellular surface. The surfaces of bacterial cells are generally negatively charged, and there are many cations, such as Mg^2+^ and Ca^2+^, to stabilize the negative charge on the cellular surfaces. These cations play crucial roles in the interaction of MVs with bacterial cells because cations can form a bridge between two negatively charged interfaces (Kadurugamuwa and Beveridge, 1996; Tashiro et al., 2010). The strong MV-cell association in *B. agrestis* was different from this case; the negative charge of the cellular surfaces in *B. agrestis* was lower than that of other bacteria, and the interaction energy between MVs and cells in this species was much lower than that in other bacteria based on the DLVO theory (**Figures 4C**). Because bacterial surface thermodynamic theory is fundamental in interpreting bacterial adhesion, several studies have applied the classic or modified DLVO theory to predict bacterial aggregation and attachment on the surface of substances (Hermansson, 1999; Strevett and Chen, 2003; Wang et al., 2013; Yoshihara et al., 2014), while it has never been used to investigate the MV-bacterial cell interaction. To our knowledge, this is the first report to apply the DLVO theory to explain the interaction between MVs and bacterial cells. It might be that interaction energy, which is characterized by hydrodynamic diameter and zeta potential, based on DLVO theory provides good indication to understand the association between MVs and cells. However, we cannot deny the possibility that factors other than interaction energy based on the DLVO theory are also involved in the specific association between MVs and bacterial cells in *Buttiauxella* spp. because the association degree of MVs from *B. agrestis* with *Buttiauxella* spp. cells was significantly higher than that with other species even when the primary maximum energies were unchanged (**Figure 5D** and Supplementary Figure S6). Bacterial cells were coated with extracellular polymeric substances (EPS) and lipopolysaccharides (LPS), and these polysaccharides play an important role in bacterial aggregation (Flemming and Wingender, 2010). MV-cell recognition was suggested to occur via LPS in *Myxococcus xanthus* because the distance between the MVs and the cellular surface was 5–10 nm, corresponding to that of the LPS (Remis et al., 2014). The factor connecting MVs and cells in *Buttiauxella* spp. remains unknown, but polysaccharides or outer membrane proteins, which are conserved in *Buttiauxella* spp., may play a role in the specific association between MVs and bacterial cells. Indeed, the proteinase K treatment decreased the MV-cell interaction in *B. agrestis* (Supplementary Figure S7), suggesting that, at least, proteins localized on the cellular surface is related to the MV-cell association.

As previously reported (Dorward et al., 1989; Kolling and Matthews, 1999; Yaron et al., 2000; Klieve et al., 2005; Chiura et al., 2011; Gaudin et al., 2013; Fulsundar et al., 2014; Ho et al., 2015), MV-mediated gene transfer also occurred in *B. agrestis* in our study (**Figure 6**). However, little is understood regarding the detailed mechanism of MV-mediated DNA transfer in any microbe. According to a previous study, DNA transfer did not occur when the recipient cells lacked genes related to natural transformation, including *comA* and *comB-F* in *A. baylyi*, indicating that the competence proteins play a role in the uptake of DNA via MVs (Fulsundar et al., 2014). Indeed, *B. agrestis* CUETM77-167 has competence-related genes, such as *comEA* and *comEC*, as determined by searching the Integrated Microbial Genomes (IMG) data warehouse (Markowitz et al., 2014), but whether these genes are related to the uptake of DNA via MVs in this strain is unknown. Notably, the plasmid contained in MVs was transferred to *E. coli* in previous reports (Kolling and Matthews, 1999; Fulsundar et al., 2014), while natural transformation was not observed in *E. coli*, suggesting that factors other than natural competence play a role in MV-mediated DNA uptake. Furthermore, we have never obtained transformants of CUETM77-167 using several transformation methods (the calcium chloride method and electroporation, as well as natural transformation), but approximately 30% of the cells received the plasmid contained in the MVs when the p-MV concentration was high. These results suggest that MV-mediated DNA transfer can be a useful method to obtain transformants in this strain. A comprehensive mechanism for DNA uptake via MVs will be a major breakthrough for developing a method of DNA incorporation through MVs.

Several previous studies have shown that MV production is increased by exposure to antibiotics, including gentamicin, polymyxin B, colistin, ceftazidime, and imipenem (Kadurugamuwa and Beveridge, 1995; Manning and Kuehn, 2011; Koning et al., 2013; Devos et al., 2015). In particular, gentamicin and antimicrobial peptides (polymyxin B and colistin) bound to LPS on bacterial cell surfaces resulted in increased MV formation (Kadurugamuwa and Beveridge, 1995; Manning and Kuehn, 2011). However, several studies have indicated that MV secretion has a protective effect against antibiotics, e.g., MVs can carry active β-lactamase (Ciofu et al., 2000; Schaar et al., 2011) or bind antibiotics in the extracellular milieu (Grenier et al., 1995; Manning and Kuehn, 2011). Thus, previous reports have focused on antibiotic resistance and the association of MVs with antibiotics; however, little is understood regarding the therapeutic potential of MVs containing antibiotics, except for several reports by the Beveridge group indicating that gentamicin-induced MVs derived from *P. aeruginosa* had a killing effect against both Gram-negative and Gram-positive bacteria (Kadurugamuwa and Beveridge, 1996; MacDonald and Beveridge, 2002). In this study, we investigated whether gentamicin-induced MVs derived from *B. agrestis* had a selective killing effect on target cells and examined the potential for the biotechnical use of MVs as antibiotic-delivery vehicles to control specific microbial species in heterogeneous microbial communities. We showed that g-MVs derived from *B. agrestis* had a higher killing effect on *B. agrestis* than on *E. coli* and *P. aeruginosa* (**Figures 7A–C**); in particular, the number of surviving *B. agrestis* cells was much lower than that of surviving *E. coli* cells in the sample including both species (**Figure 7D**). The results obtained in this study only demonstrate the effective killing of *B. agrestis*, but further comprehensive analysis of the mechanism underlying the specific association of MVs with bacterial cells, other than interaction energy based on surface charges, will create a new path for developing MVs as a tool to eliminate pathogenic or other bacterial species in heterogeneous microbial communities.

## Conclusion

We presented the first example of the delivery of cargo in MVs to specific cells. MVs derived from *B. agrestis* CUETM77-167 specifically interacted with bacterial cells of *Buttiauxella* spp. The specific interaction between MVs and *Buttiauxella* spp. cells was explained in terms of interaction energy based on the DLVO theory, but other as-yet-unknown factors may be involved in this specific interaction. Interaction of MVs with bacterial cells enabled MV-mediated gene transfer. Moreover, the specific interaction of MVs enables their use for the delivery of gentamicin to target cells. These results indicate that MVs selectively associate with bacterial cells and provide a novel model in which MVs effectively deliver their cargo to target microbial cells in microbial communities. We anticipate that studies of MVs will be a valuable contribution to the development of a novel biotechnological tool for controlling target bacterial cells.

## Author Contributions

YT and YH planned and designed the experiments. YT, YH, MS, and KT performed the experiments. YT, YH, MS, KT, MO, KK, and HF analyzed the data. YT and YH wrote the manuscript. All of the authors contributed to the discussion and provided comments on the manuscript.

## Conflict of Interest Statement

The authors declare that the research was conducted in the absence of any commercial or financial relationships that could be construed as a potential conflict of interest.
