# Supplementary material for: Interaction of Bacterial Membrane Vesicles with Specific Species and Their Potential for Delivery to Target Cells
Source: Front Microbiol. 2017 Apr 7;8:571. doi: 10.3389/fmicb.2017.00571 (PMC5383704; doi:10.3389/fmicb.2017.00571)
Supplement: Supplementary file 1 [file Data_Sheet_1.pdf]

SUPPLEMENTARY INFORMATION

Interaction of Bacterial Membrane Vesicles with Specific Species and Their Potential for Delivery to Target Cells.

Yosuke Tashiro[1]*, Yusuke Hasegawa[1], Masaki Shintani[1,2,3], Kotaro Takaki[4], Moriya Ohkuma[3], Kazuhide Kimbara[1,2] and Hiroyuki Futamata[1,2,5]

[1]*Applied Chemistry and Biochemical Engineering Course, Department of Engineering, Graduate School of Integrated Science and Technology, Shizuoka University, Hamamatsu, Japan,* [2]*Graduate School of Science and Technology, Shizuoka University, Hamamatsu, Japan,* [3]*Japan Collection of Microorganisms, RIKEN BioResource Center, Tsukuba, Japan,* [4]*Department of Applied Chemistry and Biochemical Engineering, Faculty of Engineering, Shizuoka University, Hamamatsu, Japan,* [5]*Research Institute of Green Science and Technology, Shizuoka University, Shizuoka, Japan*

***Correspondence:**
Yosuke Tashiro
tashiro.yosuke@shizuoka.ac.jp

Supplementary Table S1. Association of MVs with each bacterial cell.[a]

	Recipient strains											
MV donor strains	*C. g.*	*M. l.*	*B. s.*	*F. j.*	*R. h.*	*R. s.*	*H. p.*	*B. a.*	*E. c.*	*E. p.*	*P. ae*	*P. al*
C. glutamicum AJ224	280	456	172	483	50	101	26	546	123	14	80	72
M. luteus JCM 1464	148	184	78	196	954	137	45	123	143	11	134	89
B. subtilis C1	1135	1852	510	606	967	701	1012	1466	152	434	156	370
F. johnsoniae JCM 8514	1549	2331	642	1962	856	1200	302	1406	162	706	108	756
R. halotolerans JCM 17536	860	1794	369	954	471	701	158	677	156	178	106	297
R. soli DS-42	703	803	206	682	396	513	137	835	170	215	119	341
H. pseudoflava GA3	2111	3652	601	2240	906	1439	303	1585	147	477	149	660
B. agrestis CUETM77-167	464	405	30	184	80	98	50	1713	153	63	121	183
E. persicina HK204	137	267	80	153	138	223	86	183	169	163	169	156
P. aeruginosa PAO1	1135	1852	510	605	967	701	1012	1465	510	434	501	370
P. alcaligenes JCM 20561	808	1110	405	1075	1135	1632	408	906	201	808	131	805

[a] MVs associated with cells were determined by relative fluorescence units of FM4-64 normalized to the cellular protein concentration (mg/mL). The data are the average of three replicates.

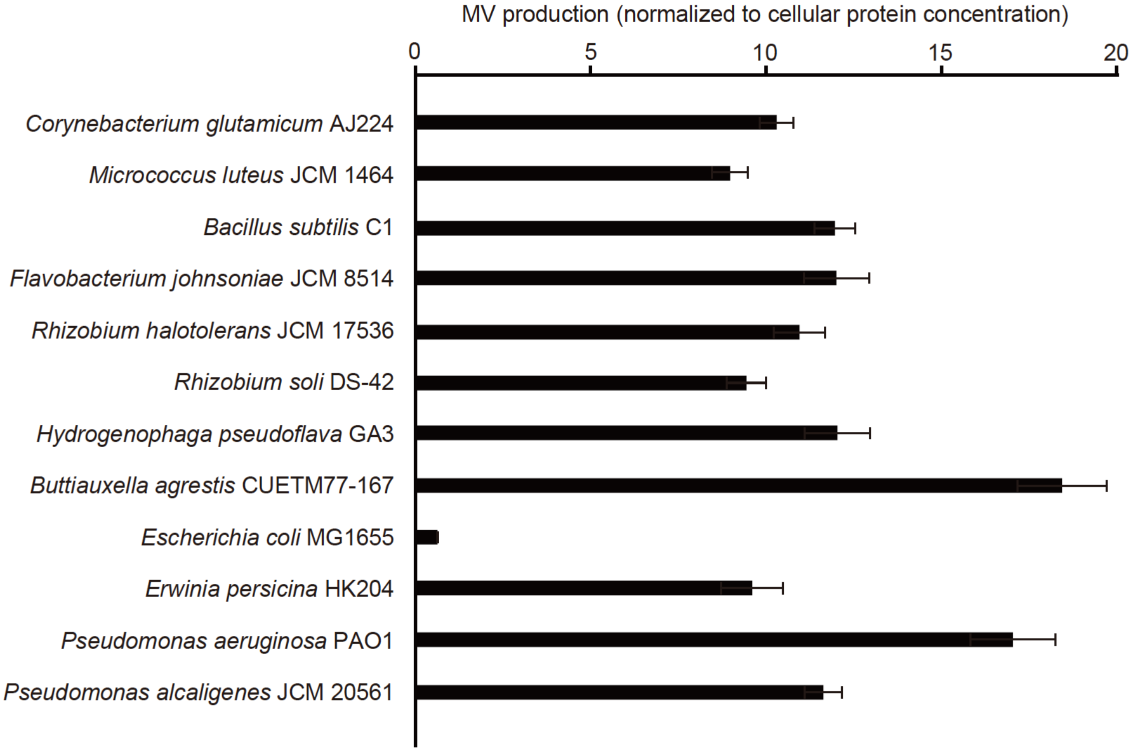

Supplementary Figure S1. Putative MV production of each microbial strain. Each strain was grown in TSB medium. The values are shown as the MV phospholipid concentration (μg/mL) in the supernatant normalized to the cellular protein concentration (mg/mL). The data are shown as the mean ± standard deviation from three replicates.

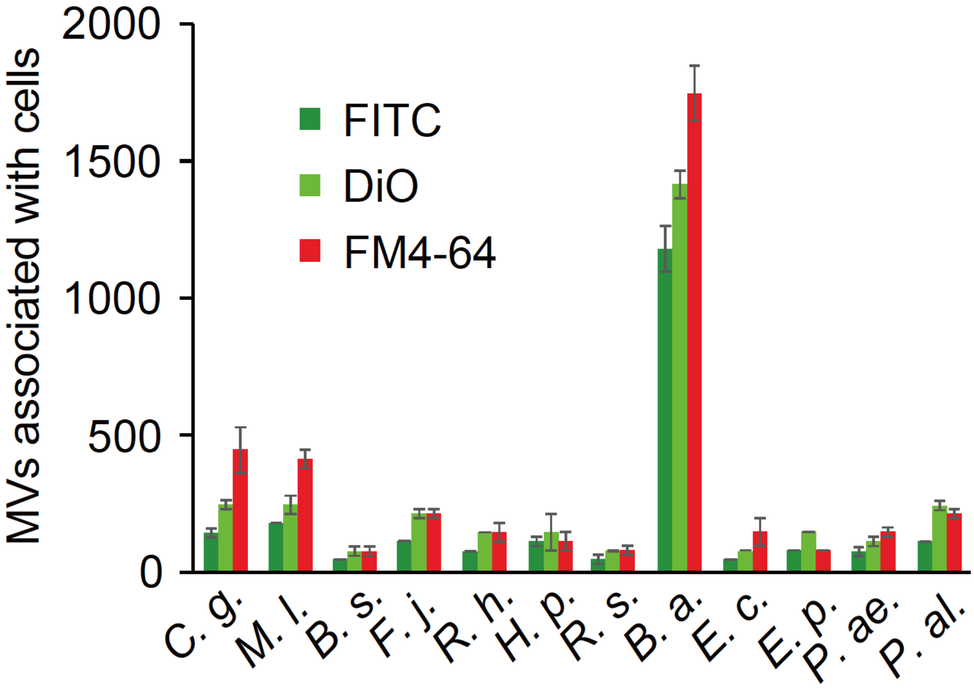

Supplementary Figure S2. Association of MVs derived from *B. agrestis* CUETM77-167 with cells of various bacterial species. MVs (20 µg/mL of phospholipids) were labeled with FITC, DiO or FM4-64 and incubated with bacterial cells for 30 min at 30°C. *Corynebacterium glutamicum* AJ2247 (*C. g.*), *Micrococcus luteus* JCM 1464 (*M. l.*), *Bacillus subtilis* C1 (*B. s.*), *Flavobacterium johnsoniae* JCM 8514 (*F. j.*), *Rhizobium halotolerans* JCM 17536 (*R. h.*), *R. soli* DS-42 (*R. s.*), *Hydrogenophaga pseudoflava* GA3 (*H. p.*), *Buttiauxella agrestis* CUETM77-167 (*B. a.*), *Escherichia coli* MG1655 (*E. c.*), *Erwinia persicina* HK204 (*E. p.*), *Pseudomonas aeruginosa* PAO1 (*P. ae.*) and *P. alcaligenes* JCM 20561 (*P. al.*) were used as the recipient strains. The MVs associated with cells were identified by relative fluorescence units (RFUs) of FM4-64 normalized to the cellular protein concentration (mg/mL). The data are shown as the mean ± standard deviation from three replicates.

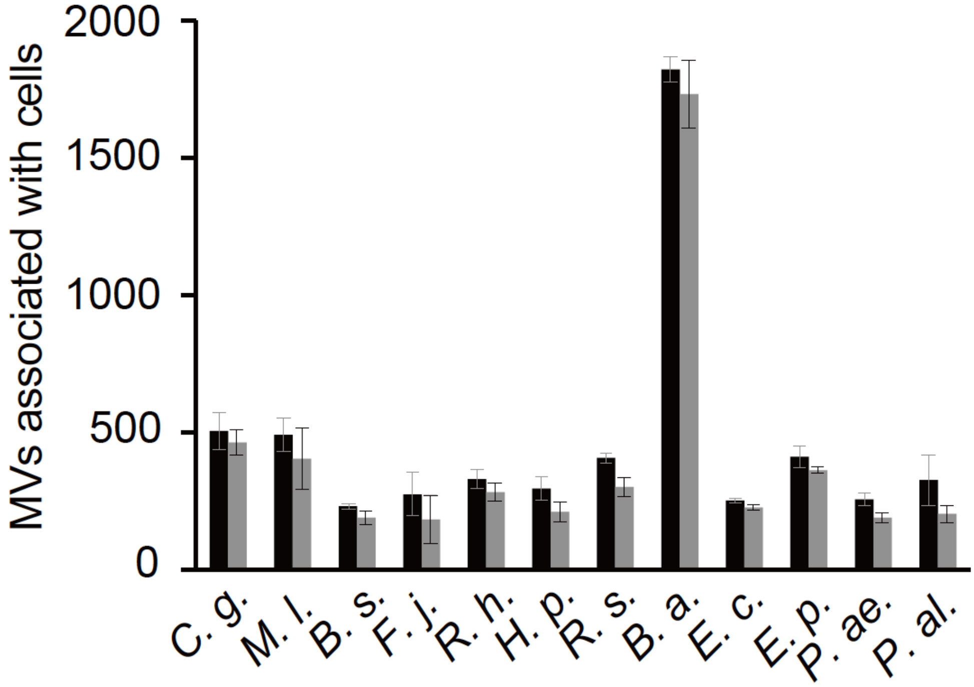

Supplementary Figure S3. Association of MVs derived from *B. agrestis* CUETM77-167 with dead (black) and live (grey) cells of various bacterial species. MVs (20 µg/mL of phospholipids) were labeled with FM4-64 and incubated with bacterial cells for 30 min at 30°C. *Corynebacterium glutamicum* AJ2247 (*C. g.*), *Micrococcus luteus* JCM 1464 (*M. l.*), *Bacillus subtilis* C1 (*B. s.*), *Flavobacterium johnsoniae* JCM 8514 (*F. j.*), *Rhizobium halotolerans* JCM 17536 (*R. h.*), *R. soli* DS-42 (*R. s.*), *Hydrogenophaga pseudoflava* GA3 (*H. p.*), *Buttiauxella agrestis* CUETM77-167 (*B. a.*), *Escherichia coli* MG1655 (*E. c.*), *Erwinia persicina* HK204 (*E. p.*), *Pseudomonas aeruginosa* PAO1 (*P. ae.*) and *P. alcaligenes* JCM 20561 (*P. al.*) were used as the recipient strains. Dead cells were prepared by exposure to UV for 30 min, and counting CFU showed that more than 99.99% of the cells in each strain lost the ability to grow. The MVs associated with cells were identified by RFUs of FM4-64 normalized to the cellular protein concentration (mg/mL). The data are shown as the mean ± standard deviation from three replicates.

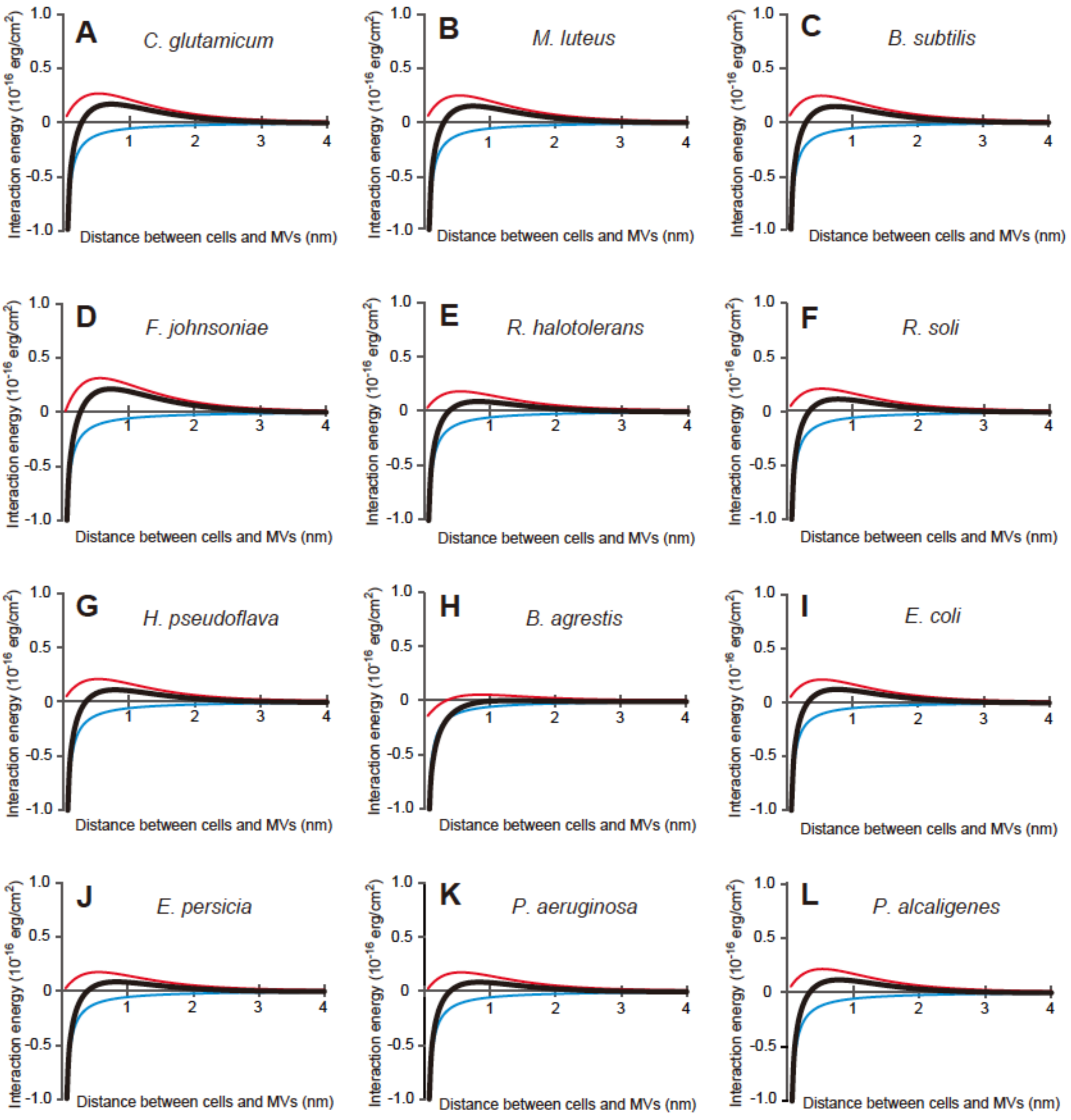

Supplementary Figure S4. Interaction energies between MVs derived from *B. agrestis* CUETM77-167 and cells of various bacterial species. The total interaction energies (black), London-van der Waals interaction energies (blue) and electric repulsive interaction energies (red) are shown. The primary maximum energies of each figure are shown in Figure 4C.

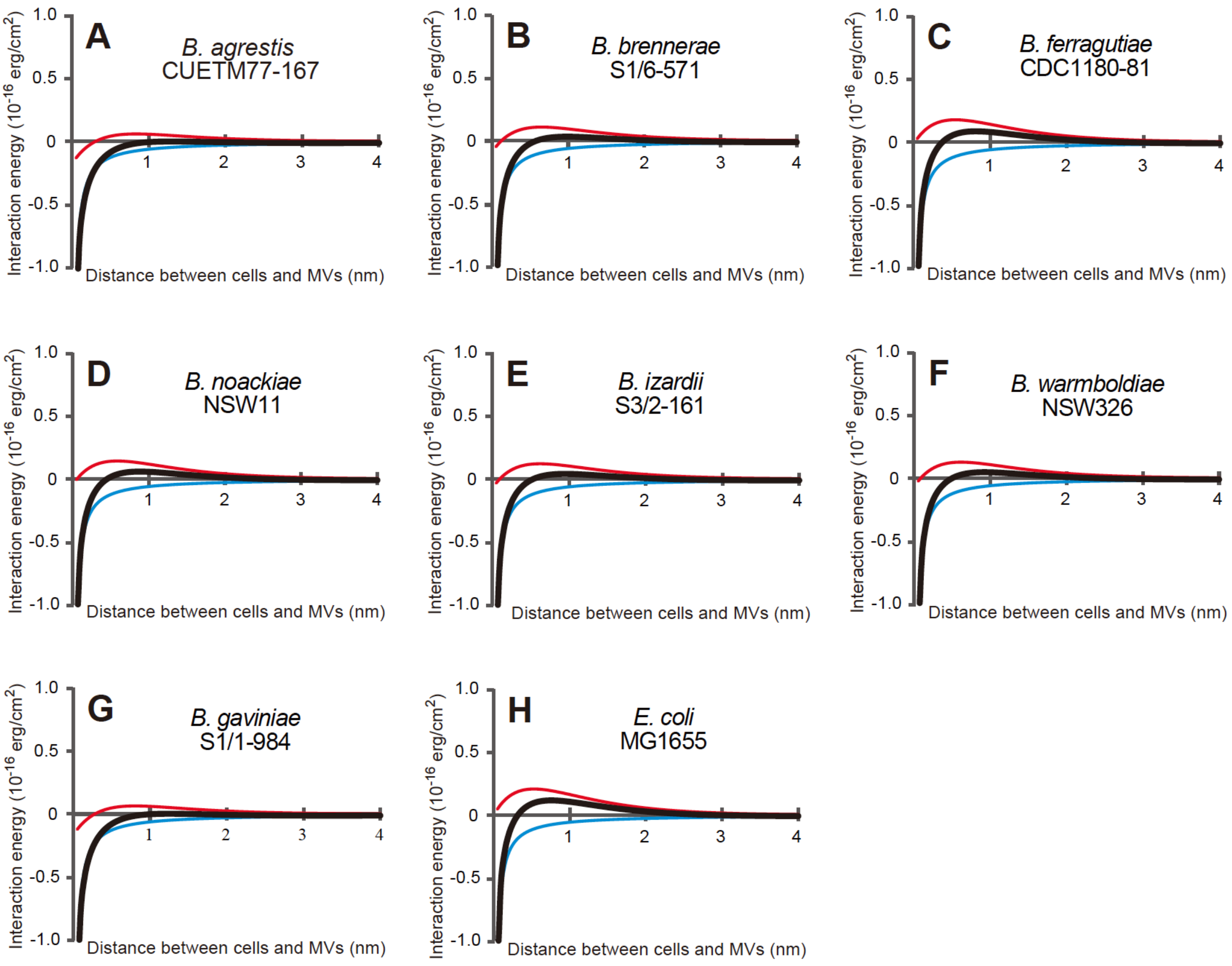

Supplementary Figure S5. Interaction energies between MVs derived from *B. agrestis* CUETM77-167 and bacterial cells belonging to *Buttiauxella* spp. The total interaction energies (black), London-van der Waals interaction energies (blue) and electric repulsive interaction energies (red) are shown. The primary maximum energies of each figure are shown in Figure 5C.

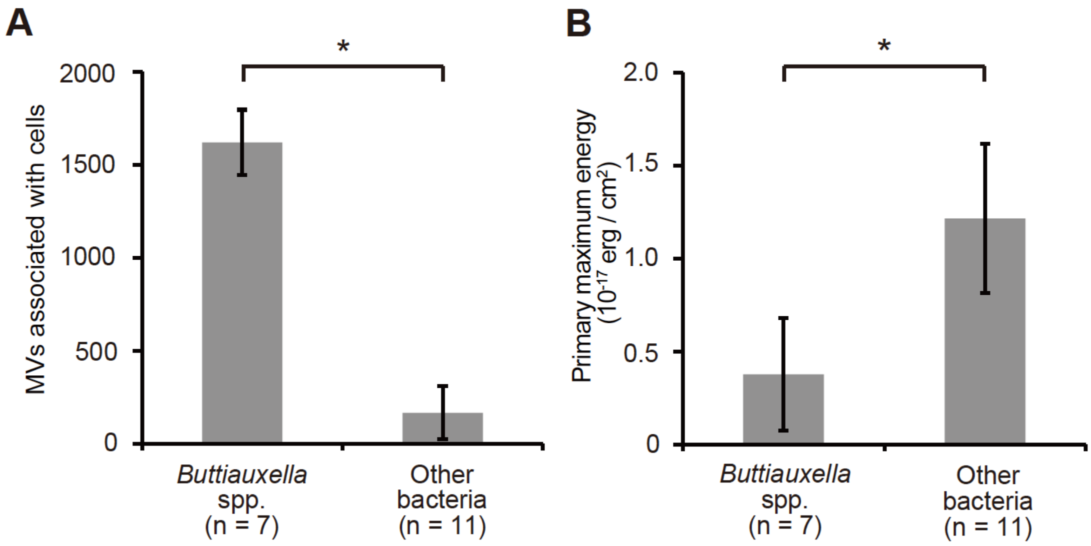

Supplementary Figure S6. Association of MVs derived from *B. agrestis* CUETM77-167 with bacterial cells (A) and the primary maximum energy between the MVs and each cell (B). The values of each figure are the same as in Figure 5D. Significant differences between the datasets are marked by asterisks ($P < 0.005$).

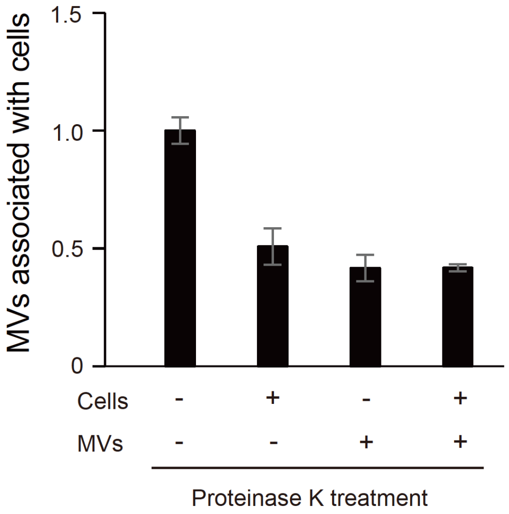

Supplementary Figure S7. Effect of proteinase K on association of MVs derived from *B. agrestis* CUETM77-167 with bacterial cells. MVs and/or cells were treated with 50 μg/mL proteinase K (+, proteinase K treatment; -, no treatment). MVs (20 μg/mL of phospholipids) derived from CUETM77-167 were labelled with FM4-64 and incubated with CUETM77-167 cells (OD_{600} = 1.0). MVs associated with cells were identified by relative fluorescence units of FM4-64 normalized to the cellular protein concentration (mg/mL). Values relative to the control (no proteinase K treatment) are shown. The data are shown as the means ± standard deviation from three replicates.
